# Effect of Amoxicillin in combination with Imipenem-Relebactam against *Mycobacterium abscessus*

**DOI:** 10.1038/s41598-020-57844-8

**Published:** 2020-01-27

**Authors:** Rose C. Lopeman, James Harrison, Daniel L. Rathbone, Maya Desai, Peter A. Lambert, Jonathan A. G. Cox

**Affiliations:** 1 School of Life and Health Sciences, Aston University, Aston Triangle, B4 7ET Birmingham, UK; 2 Birmingham Children’s Hospital, Birmingham Women’s and Children’s NHS Foundation Trust, Steelhouse Lane, B4 6NH Birmingham, UK

**Keywords:** Antibiotics, Enzymes

## Abstract

Infections caused by *Mycobacterium abscessus* are increasing in prevalence in cystic fibrosis patients. This opportunistic pathogen′s intrinsic resistance to most antibiotics has perpetuated an urgent demand for new, more effective therapeutic interventions. Here we report a prospective advance in the treatment of *M. abscessus* infection; increasing the susceptibility of the organism to amoxicillin, by repurposing the β-lactamase inhibitor, relebactam, in combination with the front line *M. abscessus* drug imipenem. We establish by multiple *in vitro* methods that this combination works synergistically to inhibit *M. abscessus*. We also show the direct competitive inhibition of the *M. abscessus* β-lactamase, Bla_Mab_, using a novel assay, which is validated kinetically using the nitrocefin reporter assay and *in silico* binding studies. Furthermore, we reverse the susceptibility by overexpressing Bla_Mab_ in *M. abscessus*, demonstrating relebactam-Bla_Mab_ target engagement. Finally, we highlight the *in vitro* efficacy of this combination against a panel of *M. abscessus* clinical isolates, revealing the therapeutic potential of the amoxicillin-imipenem-relebactam combination.

## Introduction

*Mycobacterium abscessus* is a rapidly growing, non-tuberculous mycobacteria (NTM) and increasingly prevalent opportunistic human pathogen. It is capable of causing pulmonary infections in patients with structural lung disorders such as cystic fibrosis (CF) and bronchiectasis as well as skin and soft tissue infections (SSTIs) in humans^1–6^. However, monitoring the incidence of *M. abscessus* infection is difficult, mainly due to incorrect or non-specific species identification (i.e. isolates being referred to as *M. chelonae/abscessus* group or simply nontuberculous mycobacteria) obscuring its true prevalence, which is estimated to be much higher than currently thought^7,8^.

As with other NTMs, *M. abscessus* is resistant to the frontline antibiotics used for tuberculosis treatment; rifampicin, ethambutol, pyrazinamide and isoniazid^9–12^. Despite reports that novel drugs developed to treat tuberculosis, such as bedaquiline and rifabutin may have some efficacy against *M. abscessus*, the paucity of available and potential treatments for this disease remains clear^13,14^. Infection with *M. abscessus* is treated with a combination therapy of amikacin, tigecycline and imipenem, supplemented with oral clarithromycin or azithromycin, if the patient’s isolate is macrolide susceptible, for 1 month. This is followed by a 12-month continuation phase comprising of nebulised amikacin, and a combination of clofazimine, linezolid, minocycline, moxifloxacin or co-trimoxaole, once the patient′s isolate has been susceptibility tested^15,16^. The ubiquitous environmental nature of *M. abscessus* may go some way to explaining the high levels of intrinsic drug resistance to most major classes of antibiotic that is observed clinically^17^. As a consequence of this, the efficacy of the current drug regime is poor and often does not successfully treat *M. abscessus* infections^16^.

*M. abscessus* expresses an endogenous class A β-lactamase (Bla_Mab_) conveying intrinsic resistance to a wide range of β-lactam antibiotics^18^. The identification of Bla_Mab_, a homolog of the *Mycobacterium tuberculosis* endogenous β-lactamase, BlaC, sparked widespread investigation into the potential of β-lactamase inhibitors to supplement the treatment of *M. abscessus* infection^18^. One of these studies demonstrated the inhibitory activity of avibactam, a non-β-lactam β-lactamase inhibitor, against Bla_Mab_ significantly improving the *in vitro* and *in vivo* activity of not only imipenem, but also amoxicillin, which often displays no activity against *M. abscessus*^19^. Avibactam is commercially available in combination with the cephalosporin ceftazidime, rather than imipenem^20^. Recently, multiple studies have been published demonstrating a number of β-lactam combinations displaying high levels of *in vitro* synergistic activity against *M. abscessus* complex^21,22^. Furthermore, one of these studies identified the *in vitro* activity of two new non-β-lactam β-lactamase inhibitors, named relebactam and vaborbactam, which demonstrated synergistic activity between imipenem and relebactam as well as meropenem and vaborbactam^23^. This is important, since imipenem is currently used as a front line drug against *M. abscessus* infection, due to imipenem being the carbapenem with the highest activity against *M. abscessus*^24^. Therefore, increasing the susceptibility of *M. abscessus* to imipenem would have substantial clinical impact.

In this study, we have expanded upon previous work and identified a significant step forward in treatment potential for *M. abscessus* infection^23^. We demonstrate the *in vitro* increase in susceptibility of the organism to the broad spectrum β-lactam antibiotic, amoxicillin, using the β-lactamase inhibitor, relebactam, in combination with imipenem. We establish synergy between amoxicillin, relebactam and imipenem, as well as activity against a range of *M. abscessus* clinical isolates. We have identified the mechanism of relebactam-Bla_Mab_ inhibition as competitive using recombinant protein in both a novel thin-layer chromatography (TLC)-based assay, as well as previously established spectrophotometric methods. Furthermore, this has been delineated using *in silico* molecular docking. This offers a considerable therapeutic potential as imipenem, a mainstay of current *M. abscessus* treatment, in combination with relebactam and cilastatin has recently been approved by the U.S. Food and Drug Administration (FDA) for use against urinary tract and intra-abdominal infections^25^. Therefore, the addition of amoxicillin in conjunction with the repurposing of relebactam alongside imipenem, to the current treatment regime has the potential to significantly improve treatment outcome.

## Results

### *In vitro* testing of amoxicillin-imipenem-relebactam on *M. abscessus*

**Figure 1:**
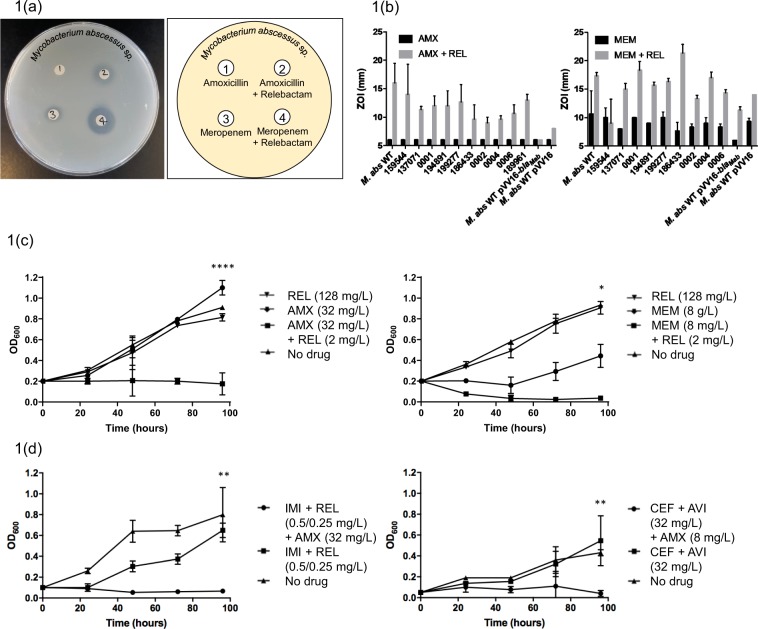
Relebactam makes *Mycobacterium abscessus* susceptible to amoxicillin and increases susceptibility to meropenem. (**1a**) A disk diffusion experiment and corresponding plate map demonstrating enhanced susceptiblity of M. abscessus (NCTC) by zone of inhibition to amoxicillin (1) with the addition of relebactam (2) and meropenem (3) with the addition of relebactam (4). (**1b**) The disk diffusion experiments were conducted with the NCTC M. abscessus strain along with a panel of clinical isolates, demonstrating enhanced susceptibility by addition of relebactam (REL) to amoxicillin (AMX) and meropenem (MEM). (**1c**) Growth curves were conducted with M. abscessus NCTC in medium containing 128 mg/L relebactam only and 32 mg/L amoxicillin with and without relebactam at 2 mg/L. Growth inhibition was only observed with relebactam in combination with amoxicillin. Likewise, the inhibitory activity of meropenem was clearly enhanced with the addition of relebactam. A t-test was used for end point analysis between samples +/− relebactam and the results were deemed to be significant with p values of <0.0001 and 0.0152 for amoxicillin and meropenem respectively. (**1d**) Growth curves were conducted with M. abscessus NCTC in medium containing 0.5 mg/L imipenem and 0.25 mg/L relebactam, with and without amoxicillin at 32 mg/L. The inhibitory activity of imipenem-relebactam was enhanced with the addition of amoxicillin (p = 0.0028). Growth curves were also conducted with M. abscessus NCTC in medium containing 32 mg/L ceftazidime and 32 mg/L avibactam, with and without amoxicillin at 8 mg/L. The inhibitory activity of ceftazidime-avibactam was enhanced by amoxicillin but to a lesser extent than imipenem-relebactam (p = 0.0016).

In this paper we have identified that *M. abscessus* can be made susceptible to amoxicillin by the addition of the competitive β-lactamase inhibitor, relebactam. We have further demonstrated that when used in conjunction with amoxicillin, the MIC of the imipenem-relebactam combination can be significantly reduced. This effect is not as pronounced in ceftazidime-avibactam in combination with amoxicillin. We also show that meropenem susceptibility can also be enhanced by the addition of relebactam. Firstly, we conducted disk diffusion assays with amoxicillin and meropenem, with and without relebactam (Fig. 1a). Subsequently the zones of inhibition (ZOI) were measured as a marker of susceptibility. Amoxicillin alone failed to demonstrate any kind of susceptibility, however the addition of relebactam provided a clear ZOI, demonstrating the induced susceptibility to amoxicillin by relebactam. A small ZOI was visible for meropenem, however with the incorporation of relebactam, this ZOI was enhanced, suggesting increased susceptibility upon combination with relebactam (Fig. 1a). In order to assess the clinical relevance of these observations, both amoxicillin and meropenem were tested alone and in combination with relebactam against a panel of clinical isolates of *M. abscessus* obtained from patients at Brighton and Sussex Medical School, in addition to *M. abscessus* NCTC strain. In all cases, the addition of relebactam resulted in the isolate becoming susceptible amoxicillin (*n* = *3*) (Fig. 1b left). The same experiment was conducted with meropenem, and in most cases, an increase in susceptibility was observed (*n* = *3*) (Fig. 1b right). All necessary controls were conducted and no ZOI was observed for relebactam alone.

In order to further validate our observation, liquid cultures were exposed to increasing concentrations of amoxicillin and meropenem with and without a dose response of relebactam (Fig. 1c). As a control, relebactam was also tested for inhibitory activity on its own. Relebactam at 128 mg/L demonstrated no inhibitory activity, giving a growth profile much the same as the compound-free control (no drug), confirming that relebactam lacks antibacterial activity against *M. abscessus*. 32 mg/L of amoxicillin appeared to show a moderate enhancement of bacterial growth, however when combined with 2 mg/L of relebactam, this amoxicillin concentration was found to display potent antibacterial activity against *M. abscessus* (Fig. 1c). The same experiment was conducted with meropenem, wherein partial growth impairment was observed at 8 mg/L, however when combined with 2 mg/L relebactam, significant antibacterial activity was observed at this concentration. End point statistical analysis was conducted using a t-test to assess the significance of differences between cultures with and without relebactam. In both cases, a statistically significant increase in susceptibility was observed upon addition of 2 mg/L of relebactam in combination with amoxicillin or meropenem.

Since relebactam is only available in combination with imipenem, experiments with amoxicillin in conjunction with imipenem-relebactam were conducted, alongside amoxicillin and ceftazidime-avibactam (Fig. 1d top and bottom respectively). Both of these β-lactam/ β lactamase inhibitor combinations are commercially approved by the FDA, however their efficacies against *M. abscessus* are yet to be compared. An experiment was conducted using a fixed ratio of imipenem-relebactam with and without amoxicillin, representative of the ratio of these drugs in the approved combination. At concentrations of 0.5:0.25 mg/L imipenem:relebactam without amoxicillin, growth was observed to be similar to that of the *M. abscessus* cells only control, whereas with the addition of 32 mg/L amoxicillin, total inhibition of growth is seen. A similar experiment was conducted using ceftazidime:avibactam with and without amoxicillin. Inhibition was observed at ceftazidime:avibactam 32:32 mg/L with the addition amoxicillin 8 mg/L, and those same concentrations with no amoxicillin were again similar to the no drug control.

*In vitro* antibiotic susceptibility testing was performed for imipenem, amoxicillin and relebactam, either on their own, in conjunction with each other, or as a triplicate combination, against 16 *M. abscessus* clinical isolates, *M. abscessus* containing the Bla_Mab_ constitutive overexpression plasmid pVV16-*bla*_Mab_ and *M. abscessus* pVV16 (Table 1). All but one isolate (93.75%) had MICs of >128 mg/L for amoxicillin and >32 mg/L for relebactam, indicating these isolates are not susceptible to these drugs within their reasonable clinical range. When combining amoxicillin with relebactam, 81.25% of the strains became susceptible to amoxicillin, with MICs ranging from 4–128 mg/L, requiring 0.5–16 mg/L of relebactam to produce this effect. The imipenem MICs ranged from 2–8 mg/L, therefore according to CLSI breakpoint guidelines, all of the clinical isolates were susceptible or intermediate to imipenem.

**Table 1:** Minimum Inhibitory Concentrations (MIC) of imipenem and/or amoxicillin in combination with relebactam against *M. abscessus* clinical isolates (including NCTC 13031) and *M. abscessus* pVV16-*bla*_Mab_ and *M abscessus* pVV16.

*M. abscessus* strain	MIC (mg/L)									
	AMX	IMI	REL	AMX (+REL)	IMI (+REL)	REL (+AMX)	REL (+IMI)	AMX (+IMI +REL)	IMI (+AMX^+^REL)	REL (+AMX +IMI)
NCTC	>128	2	>32	64	2	2	1	**32**	**0.5**	**0.25**
DC088 A	>128	4	>32	128	2	>32	1	**64**	**1**	**0.5**
DC088 B	>128	4	>32	32	4	>32	2	**32**	**1**	**0.5**
DC088 C	>128	8	>32	>128	4	>32	2	**32**	**1**	**0.5**
DC088 D	>128	4	>32	>128	4	>32	2	**4**	**2**	**1**
DC088 E	>128	2	>32	128	2	16	1	**64**	**1**	**0.5**
DC088 Ref	>128	4	>32	>128	2	>32	1	**64**	**1**	**0.5**
211666	>128	4	>32	128	2	8	1	**64**	**1**	**0.5**
137071	>128	2	>32	128	4	16	2	**16**	**2**	**1**
199277	>128	4	>32	64	4	16	2	**128**	**1**	**0.5**
194891	8	4	>32	4	2	0.5	1	**64**	**1**	**0.5**
159544	>128	4	>32	128	2	1	1	**8**	**1**	**0.5**
186433	>128	2	>32	128	4	32	2	**128**	**1**	**0.5**
186144	>128	8	>32	128	4	4	2	**64**	**1**	**0.5**
186154	>128	16	>32	>128	4	>32	2	**8**	**4**	**2**
147028	>128	4	>32	>128	4	>32	2	**16**	**1**	**0.5**
pVV16-*bla*_Mab_	>128	32	>32	128	8	16	4	**0**	**2**	**4**
pVV16	>128	2	>32	32	2	2	1	**32**	**1**	**0.5**

*M. abscessus* pVV16-*bla*_Mab_ had an MIC of imipenem of 32 mg/L. When combining imipenem with relebactam (MIC range 1–2 mg/L), the MICs of imipenem were reduced 2-fold in 43.75% of the clinical isolates, produced no effect in 31.25% of the clinical isolates and surprisingly, increased the MIC of imipenem 2-fold in 6.25% of the clinical isolates. The MIC of imipenem against *M. abscessus* pVV16-*bla*_Mab_ was reduced 4-fold from 32 mg/L to 8 mg/L with the addition of 4 mg/L relebactam. The MIC of imipenem against *M. abscessus* pVV16 was unchanged at 2 mg/L. When imipenem and relebactam (MIC range 0.25–1 mg/L) were combined with amoxicillin (MIC range 8–128 mg/L), the MICs of imipenem reduced 2-fold in 18.75% of the clinical isolates, 4-fold in 62.5% of the clinical isolates, 8-fold in 12.5% of the clinical isolates, remained the same in 6.25% of the clinical isolates and reduced 16-fold in *M. abscessus* pVV16-*bla*_Mab_ compared with imipenem alone. Briefly, the MIC ranges for imipenem alone in our isolates was 2–8 mg/L, which was reduced to 2–4 mg/L with the addition of relebactam (aside from *M. abscessus* pVV16-*bla*_Mab_ which has an MIC of 8 mg/L), and further reduced to 0.5–4 mg/L with the addition of both relebactam and amoxicillin. The MIC ranges for amoxicillin were generally higher and more variable than those of imipenem, with an MIC range of 4–128 mg/L with the addition of relebactam, with some isolates lacking susceptibility at the highest concentration tested. The addition of both relebactam and imipenem resulted in an MIC range of 4–128 mg/L. This result is replicated using the ZOI method, which clearly demonstrated drug-target interaction between relebactam and Bla_Mab_, as no activity of amoxicillin is seen. Plate images and plate map for the subsequent resistance induced by Bla_Mab_ overexpression are shown in Supplementary Figure 1.

Although 93.75% of *M. abscessus* clinical isolates are resistant to amoxicillin on its own, with MICs of >128 mg/L, the results in Table 1 show that amoxicillin susceptibility in *M. abscessus* isolates can be seen with the addition of the β-lactam inhibitor, relebactam. It is also well established that imipenem is effective against most strains of *M. abscessus*, but the results from Table 1 clearly show that the efficacy of imipenem can be enhanced with the addition of relebactam, and further enhanced with the addition of relebactam and amoxicillin.

### Biochemical analysis of relebactam inhibition of *M. abscessus* β-lactamase

**Figure 2:**
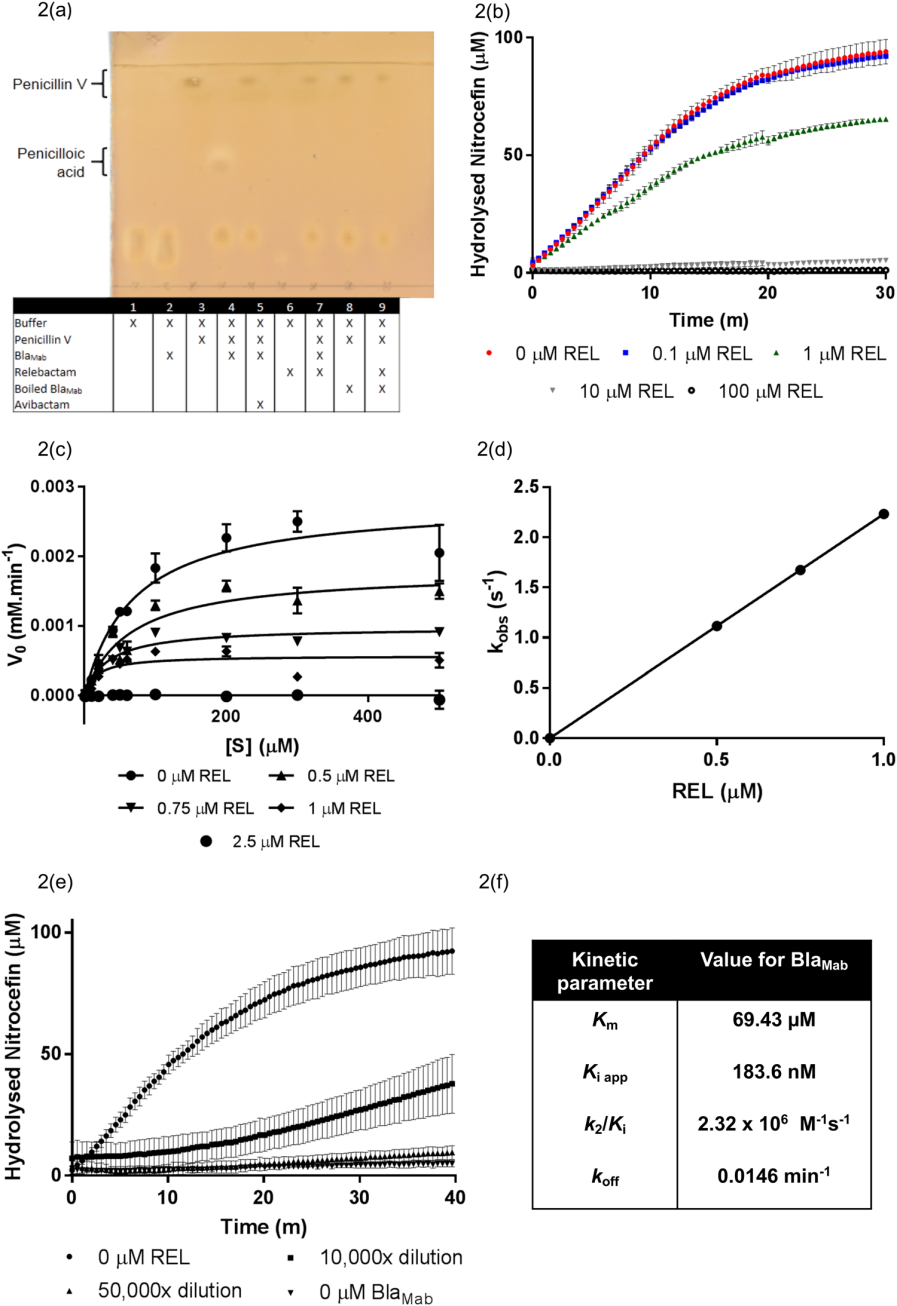
Biochemical analysis of relebactam inhibition of *M. abscessus* $\beta$-lactamase, Bla_Mab_. (**2a**) Our novel Thin Layer Chromatography (TLC) assay exhibiting the activity of BlaMab in the turnover of penicillin V (high Rf value) to penicilloic acid (lower Rf value). In the absence, or termination of activity of BlaMab (by boiling (100 °C) for 1 h) or addition of known inhibitor avibactam^19,27^ (200 µg/mL) no lower Rf value spot corresponding to penicilloic acid is resolved by TLC. The addition of relebactam to the reaction between BlaMab and penicillin V also results in the absence of the lower Rf value spot. (**2b**) This observed inhibition is validated by a spectrophotometric analysis. The increase in concentration of relebactam resulted in partial inhibition of nitrocefin turnover at 1 µM and total abrogation at 10 µM. The initial velocity (vi) of the reaction between BlaMab and nitrocefin was monitored for a range of substrate concentrations (1–500 µM) and relebactam concentrations (0, 0.5, 0.75, 1 and 2.5 µM). (**2c**) This data was plotted vi vs [S] in order to determine Km values. (**2d**) The values for kobs were obtained as previously described^19^ and plotted against relebactam concentrations ([I]) to deduce a carbamylation rate (k2/Ki) for BlaMab. (**2e**) The kinetics of BlaMab decarbamylation were assessed to show the recovery of nitrocefin hydrolysis by BlaMab after inhibition by relebactam in order to derive a koff value. (**2 f**) Kinetic parameters were derived as described previously^19^.

In order to validate the inhibitory activity observed phenotypically in Fig. 1, we conducted biochemical analysis of the activity of relebactam on the *M. abscessus* endogenous β-lactamase, Bla_Mab_. The gene was amplified by PCR, digested, ligated into pET28a and sequenced, before transformation into chemically competent *E. coli* BL21. Bla_Mab_ was expressed, cells harvested and the enzyme purified by IMAC. We subsequently devised a novel TLC-based assay for assessing β-lactamase activity by separating the penicillin V substrate from the penicilloic acid product. This assay enabled us to demonstrate the β-lactamase activity of our purified Bla_Mab_ (Fig. 2a), as well as assay the efficacy of inhibitors against the enzyme (Supplementary Fig. 2a,b). We used avibactam as a positive control, as its inhibitory activity against Bla_Mab_ has previously been described by Dubée *et al*.^19^. Lane 1 contained protein purification buffer only, and lane 2 had the addition of enzyme (0.01 mg/mL). Following chromatography, two lower spots are observed in lanes 1 and 2, indicative of buffer. Penicillin V was added to lane 3 and gave a characteristic spot of high R_f_ value, demonstrating unhydrolysed penicillin resulted in a spot just below the solvent front. Lane 4 was identical to lane 3 with the addition of Bla_Mab_ protein. The hydrolysis of penicillin V to penicilloic acid by Bla_Mab_ resulted in a spot with reduced R_f_ value. The pre-incubation of enzyme with avibactam in lane 5 resulted in a loss of the lower penicilloic acid spot, demonstrating the inhibition of Bla_Mab_ activity. In lane 6, relebactam alone did not resolve on the TLC, but its pre-incubation with Bla_Mab_ before addition to the penicillin substrate in lane 7 resulted in total inhibition of hydrolysis as observed with avibactam (lane 5). Finally, the inhibition of Bla_Mab_ is further validated by the repeat of lane 4 and lane 7 conditions with heat-denatured Bla_Mab_ (lanes 8 and 9 respectively). This result confirms the direct inhibition of Bla_Mab_ by relebactam (*n* = *5*).

We investigated the kinetic parameters of the Michaelis-Menten kinetics, the apparent *K*_i_ (*K*_i app_), the second-order carbamylation rate constant (*k*_2_/*K*_i_) and the decarbamylation rate constant (*k*_off_) of relebactam inhibition of soluble recombinant Bla_Mab_ using a commercially available β-lactamase substrate, nitrocefin, as a reporter substrate. Nitrocefin is selectively hydrolysed by β-lactamases, resulting in an increase in absorbance which can be monitored at 486 nm. By pre-incubating Bla_Mab_ enzyme with a dose response of relebactam (from 0–100 μM), before initiation of the absorbance assay with the addition of nitrocefin substrate, we observed partial inhibition at 1 μM (0.348 μg/mL) and a complete loss of activity at 10 μM (3.48 μg/mL), confirming the direct inhibitory activity of relebactam on Bla_Mab_ (Fig. 2b).

The initial velocities (v_i_) of nitrocefin turnover were recorded for a range of nitrocefin concentrations (1–500 µM) over a range of relebactam concentrations (0–2.5 µM). These results were plotted as classical Michaelis-Menten curves (v_i_ vs [S]) (Fig. 2c), resulting in Michaelis constants (*K*_m_) between 69.43–16.96 µM, before initial velocities were abolished at 2.5 µM relebactam. However, we were unable to derive a *K*_i_ value using this data. In response, we plotted the reciprocal initial velocities against relebactam concentrations as a linear equation and derived *K*_i app_ observed from the Y intercept/slope, which was then normalised for the use of nitrocefin (Fig. 2f)^26^. The *K*_i app_ value obtained of 183.6 nM is indicative of the high inhibitory potency of relebactam against Bla_Mab_. The carbamylation rate constant (*k*_2_/*K*_i_) of 2.32 ×10^6^ M^−1^s^−1^ is similar to the observed rate for avibactam inhibition of Bla_Mab_ (4.9 ×10^5^ M^−1^s^−1^)^20^. The rate of decarbamylation (*k*_off_) of Bla_Mab_ for relebactam was 0.0146 min^−1^, which is again similar to the rate previously obtained for that of avibactam (0.047 min^−1^)^19^. Our kinetics analysis show that, like avibactam, relebactam is a potent, competitive and reversible inhibitor of Bla_Mab_, displaying a reasonably rapid “on” rate and slow “off” rate, with only half the enzyme recovering activity after 40 minutes in the absence of relebactam (Fig. 2e)^19^.

Our TLC-based β-lactamase assay enabled us to further explore the parameters of the inhibitory activity of relebactam by varying the time of pre-incubation of relebactam with Bla_Mab_ (Supplementary Fig. 2a) and the minimum inhibitory concentration required to abrogate catalytic turnover of the penicillin V substrate to the penicilloic acid product (Supplementary Fig. 2b). We found that penicillin V turnover was rapid and that only addition of relebactam at the same time as the substrate demonstrated turnover of penicillin V, suggesting competitive, reversible inhibition of Bla_Mab_ by relebactam. The dose response of relebactam demonstrated total inhibition down to 20 µg/mL, and activity of Bla_Mab_ was maintained below a relebactam concentration of 2 µg/mL. This suggested a minimal concentration of relebactam required to inhibit Bla_Mab_ in the assay is 20 µg/mL, which corresponds to a less than or equal to 100 fold stoichiometric excess of relebactam required to completely inhibit Bla_Mab_ (0.5 µM BlaMab to 57.5 µM relebactam corresponding to 20 µg/mL).

### *In silico* analysis of relebactam binding to *M. abscessus* β-lactamase

**Figure 3:**
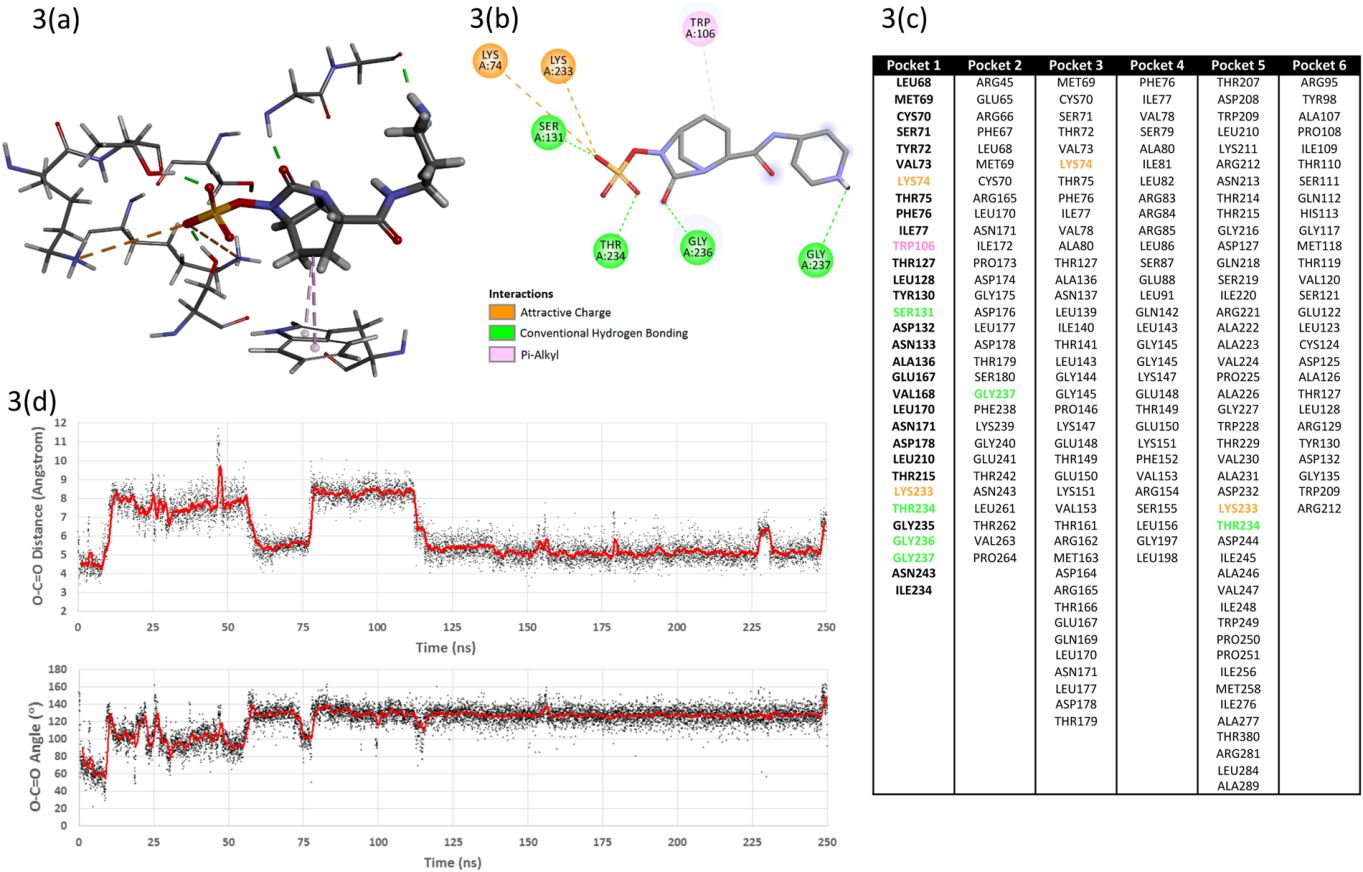
*In silico* modelling of the possible interaction of relebactam with the *M. abscessus* β-lactamase, Bla_Mab_. (**3a** and **3b**) 3D and 2D protein-ligand interaction diagrams for relebactam in the main (catalytic) active site after molecular dynamics simulation. Amino acid residues featured in the top six potential binding pockets identified for BlaMab. (**3c**) Pocket 1 corresponded to the main (catalytic site) in the enzyme and for the purposes of the docking experiment was redefined as all amino acid residues within 8 Å of serine 71. Time-courses of the serine 71 hydroxyl oxygen – relebactam carbonyl carbon distance and the corresponding O-C=O angle. (**3d**) The actual values are plotted as black points and a 50-frame moving average is over-plotted in red.﻿

In order to further investigate the mechanism of relebactam inhibition of Bla_Mab_, we conducted molecular docking simulations *in silico*. 6 potential binding sites were identified (Fig. 3c). For pockets 2–6 the ligand was weakly-held and generally exited the pocket after a few tens of nanoseconds. For pocket 1 (corresponding to the main active site) the ligand reoriented itself relative to the docked conformation and thereafter remained relatively stable within the pocket. The binding interactions for the stable pose are shown in Fig. 3(a,b) and include several polar interactions with the sulphonamide moiety and hydrophobic interactions between the relebactam central piperidine ring and tryptophan 106. In addition, after the initial reorientation, the relebactam carbonyl carbon remained in the vicinity of the hydroxyl oxygen of the catalytically-active serine 71 as can be seen after 120 ns in the distance plot given in Fig. 3(d). The average distance in this period was approximately 5.5 Å and there were many close approaches. Furthermore, the corresponding O-C=O angle (Fig. 3d) in the same period was roughly 130° and as such it is reasonable to assume that it would be possible for the serine hydroxyl to attack the relebactam carbonyl and effect a ring-opening. A further molecular dynamics simulation of the enzyme in a periodic box of explicit water was undertaken but this time in the presence of ten copies of unbound relebactam. Over the course of a 200 ns simulation, one ligand found its way into the main active site (pocket 1) after 130 ns and remained stable therein. The other nine copies of the ligand found no place to reside in the enzyme.

## Discussion

In this paper we have demonstrated the inhibitory activity of the non-β-lactam based β-lactamase inhibitor, relebactam, against the endogenous *M. abscessus* β-lactamase, Bla_Mab_, resulting in increased susceptibility of the organism to β-lactam activity. Inhibition of Bla_Mab_ only is not sufficient to kill *M. abscessus*, but this does open up the ability to utilise a wider range of β-lactam antibiotics, therefore the use of relebactam as part of a drug combination represents considerable therapeutic potential. We have identified that a low concentration of relebactam co-administration is capable of making *M. abscessus* NCTC and clinical isolates susceptible to amoxicillin, well within the therapeutic range for this versatile and widely available antibiotic. Relebactam has recently been approved by the FDA for administration in combination with imipenem, a mainstay in *M. abscessus* front line chemotherapy, and cilastatin. In conjunction with imipenem, the concentration of relebactam and amoxicillin required to combat *M. abscessus* are reduced further. Meropenem was not utilised within this triplicate compound combination, since imipenem has been previously shown to be the most effective carbapenem against *M. abscessus*, whereas amoxicillin has a broader range of activity against penicillin-binding proteins^24^.

Despite being an important component of *M. abscessus* treatment, imipenem is considered a last-resort antibiotic and is associated with severe side effects and toxicity, so it is therefore in the patients interest to provide the lowest possible dosage^28^. The addition of amoxicillin reduces the concentration of imipenem-relebactam needed to observe an inhibitory effect by 4-fold, indicating its potential as a combination therapy that maximises the inhibitory effect, whilst reducing the superfluous chemotherapeutic burden to the patient. Interestingly, the same could not be observed with the ceftazidime-avibactam combination. Whilst the addition of amoxicillin enhanced the inhibitory effect, relatively higher concentrations of ceftazidime-avibactam are needed, reducing its therapeutic potential as combination therapy against *M. abscessus* disease. This is of note, since relebactam is a derivative of avibactam, the difference in inhibition efficacy is unusual^29^. This is possibly due to the additive effect between amoxicillin and imipenem alongside relebactam aiding in reducing the overall inhibitory concentrations, whereas the combination of ceftazidime-avibactam is less potent since *M. abscessus* is more resistant to ceftazidime than imipenem, reducing this additive effect^22^.

The data in Table 1 shows that across all clinical isolates that were examined, the concentrations of both amoxicillin and imipenem required to inhibit bacterial growth were significantly reduced upon the addition of relebactam. This suggests that all clinical isolates are expressing Bla_Mab_ endogenously, despite a single isolate (194891) showing a higher susceptibility to amoxicillin before addition of relebactam, suggesting this isolate has a lower expression level than the others. The combination of all three drugs resulted in an additional reduction in concentration of amoxicillin, imipenem and relebactam repespectively. The data also shows that the overexpression of Bla_Mab_ results in the MIC of both amoxicillin and imipenem increasing, and that, in turn, higher concentrations of relebactam are required to overcome this synthetic resistance. This clearly demonstrates whole cell target engagement and inhibition of Bla_Mab_ by relebactam. Previous research, which assessed the concentrations of these compounds that can be achieved in the epithelial lining fluid (ELF) of alveoli, suggests that the MICs reported in Table 1 would be able to be reached during treatment of patients^30^. In a phase I trial, the ELF concentrations of relebactam and imipenem achieved after multiple dose treatment (at 250 mg REL and 500 mg IMI) have been reported to be 15 µM (5.23 mg/L) and 32 µM (9.58 mg/L) respectively, which are well above the MICs required to inhibit *M. abscessus* growth reported in Table 1^30^.

Our study introduces a novel TLC-based $\beta$-lactamase inhibition assay, validated with the commercially available and widely used nitrocefin assay, to investigate the parameters of relebactam′s inhibitory activity. The results of both the kinetics analysis and the *in silico* binding studies demonstrate the mechanism of inhibition of Bla_Mab_ by relebactam as competitive reversible, displaying a reasonably rapid “on” rate and slow “off” rate, indicative of the high potency of the inhibitor against Bla_Mab_.

Our findings represent a timely and highly impactful discovery that could be easily translatable into the clinical setting, providing a new therapeutic option for *M. abscessus* infections.

## Materials and Methods

### Bacterial isolates

A total of 16 *M. abscessus* clinical isolates from Brighton and Sussex Medical School and *M. abscessus* NCTC 13031 were used in this study. Stock solutions of the isolates were kept in 50% glycerol (Sigma, Dorset, UK) and Middlebrook 7H9 Broth and stored at −80 °C. Isolates were grown in Middlebrook 7H9 medium supplemented with 10% oleic acid-albumin-dextrose-catalase (OADC), 1% glycerol (50% w/v) and 0.05% Tween 80 (v/v) prior to testing. *Escherichia coli* Top 10 cells were used for propagation of plasmid DNA. These cells were routinely grown in nutrient broth, or nutrient agar (Oxoid, UK) at 37 °C. *E. coli* BL21 (DE3) cells were used for the overproduction of recombinant protein, grown in Terrific Broth (Melford, UK) at 37 °C.

### Antimicrobials

The antimicrobial agents meropenem (MEM), amoxicillin (AMX) and phenoxymethylpenicillin (Penicillin V/PenV) were obtained from Sigma Aldrich (Dorset, UK) and relebactam (REL), imipenem (IMI) and avibactam (AVI) were obtained from Carbosynth (Compton, UK). Stock solutions were prepared in sterile de-ionised water and stored at −20 °C until use.

### Disk diffusions

For the disk diffusion assays, *M. abscessus* clinical isolates were grown in Middlebrook 7H9 Broth to logarithmic phase and 100 µL of bacterial culture was inoculated into 10 mL Middlebrook 7H9 Broth supplemented with 0.7% bacteriological agar. This was poured as a layer in agar plates on top of 15 mL Middlebrook 7H11 Agar supplemented with 10% OADC and 1% glycerol. 6 mm sterile filter paper diffusion disks were placed on the agar and were subsequently impregnated with antibiotic at the following concentrations in sterile distilled water: relebactam 1 µL of 10 mg/mL, amoxicillin 3.3 µL of 3 mg/mL and meropenem 1 µL of 10 mg/mL. Plates were incubated at 30 °C for 5 days or until clear zones of inhibition were visualised. Zones of inhibition were measured across the diameter to include the disk itself in mm. Disk diffusions were also performed on *M. abscessus* pVV16-*bla*_Mab_ and *M. abscessus* pVV16.

### Broth microdilution assay

The broth microdilution assay was performed as described previously with alterations appropriate to this study^31,32^. Plates were prepared by serially diluting amoxicillin (128 mg/L–0 mg/L) or meropenem (128 mg/L–0 mg/L) in the *x*-axis and either relebactam (32 mg/L–0 mg/L) or relebactam: imipenem (32: 64 mg/L–0 mg/L) or avibactam: ceftazidime (128: 128 mg/L–0 mg/L) in the *y-*axis. 80 µL of *M. abscessus* NCTC 13031 suspension adjusted to an OD_600_ of 0.1–0.2 was inoculated in each well to reach a final well volume of 100 µL. Assay plates containing amoxicillin and/or imipenem with or without relebactam were also tested against 15 other clinical isolates and *M. abscessus* pVV16 *bla*_Mab_ and *M. abscessus* pVV16.

Plates were sealed and incubated at 30 °C for 3–5 days. The minimum inhibitory concentrations (MICs) of the various combinations were determined by optical density measurement using a spectrophotometric plate reader and identified as the well that had the lowest concentrations of both compounds and exhibited no bacterial growth (for imipenem susceptibility testing, CLSI breakpoints were utilised (Institute, Clinical and Laboratory Standards 2012). The relevant well was retroactively plotted into a growth curve over time, and this growth curve was compared to the wells containing no drug, and the wells containing either relebactam, relebactam-imipenem or ceftazidime-avibactam only and amoxicillin only. The broth microdilution assay was also performed on *M. abscessus* pVV16-*bla*_Mab_ and *M. abscessus* pVV16.

### Generation of *M. abscessus* pVV16-*bla*_*Mab*_ overexpressor strain

The *bla*_*Mab*_ gene (MAB_2875) from *M. abscessus* NCTC 13031 was amplified by polymerase chain reaction (PCR). Primers used were as follows (with restriction site underlined): Forward primer AAAAAAGGATCCGCGCCGGACGAACTCGCC and Reverse primer AAAAAAAAGCTTAGCGCCGAAGGCCCGCAG (Eurofins Genomics). Amplicons were purified and cloned into pVV16 using *Bam*HI/*Hin*DIII restriction sites and the correct sequence was confirmed by DNA sequencing (Eurofins Genomics). Both the plasmid pVV16 and the construct pVV16-*bla*_*Mab*_ were inserted into *M. abscessus* NCTC 13031 cells by electroporation (2.5 kV, 25 µF and 1000 Ω).

### Expression and purification of recombinant Bla_Mab_

The *bla*_*Mab*_ gene, with the first 90 base pairs omitted (resulting in a −30 residue N-terminal truncated protein), was amplified by PCR using the following primers (with restriction sites underlined): Forward primer AAAAAAGGATCCGCGCCGGACGAACTCGCC and Reverse primer AAAAAAAAGCTTTCAAGCGCCGAAGGCCCG (Eurofins Genomics). Amplicons were purified and cloned into pET28a using *Bam*HI/*Hin*DIII restriction sites and the correct sequence was confirmed by DNA sequencing (Eurofins Genomics). Bla_Mab_ was expressed in *E. coli* BL21 (DE3) cells by addition of 1 mM Isopropyl β-D-1-thiogalactopyranoside (IPTG) and incubation at 25 °C for 18 h. Bla_Mab_ (6×-His tagged) was purified by Ni^2+^ Immobilised Metal Affinity Chromatography (IMAC) and dialysed into 25 mM Tris HCl pH 7, 100 mM NaCl.

### Thin Layer Chromatography (TLC) activity assay

Relebactam (2 mg/mL) was added to recombinant Bla_Mab_ (0.01 mg/mL) and incubated for 5 min at room temperature, before addition of Penicillin V (4 mg/mL) for a further 10 min incubation at room temperature. Alongside appropriate control reactions (Fig. 2a), 1 µL of the reaction was spotted onto aluminium backed silica gel plates (5735 silica gel 60 F_254_, Merck) and dried before being subjected to Thin Layer Chromatography (TLC) using ethyl acetate:water:acetic acid (C_4_H_8_O_2_:H_2_O:CH_3_COOH) (3:1:1, v/v/v). Once dry, plates were visualised by being dipped into KMnO_4_ TLC stain with light charring.

### Biochemical analysis of Bla_Mab_ inhibition by relebactam

Recombinant Bla_Mab_ (0.25 nM) was mixed with an increasing concentration of relebactam (0, 0.1, 1, 10 and 100 µM) and 100 µM nitrocefin (Carbosynth, UK). The hydrolysis of nitrocefin was monitored at 486 nm using a Multiskan Go plate reader (Thermo Scientific). This was repeated using a varying concentration of nitrocefin (1–500 µM) with a shorter range of relebactam concentrations (0, 0.5, 0.75, 1 and 2.5 µM) and initial velocities (v_i_) were plotted against substrate concentration. Data analysis was performed as previously described^22,26^. Data analysis was conducted using Graphpad Prism 7.

The decarbamylation rate was determined by incubation of Bla_Mab_ (1 µM) with relebactam (20 µM) for 1 hour at 25 °C. The reaction mixture was then subsequently diluted both 10,000 and 50,000 fold (1.8 nM and 0.36 nM relebactam with 90 pM and 18 pM Bla_Mab_ respectively), before addition of 100 µM nitrocefin. The reaction was monitored at 486 nm using a Multiskan Go plate reader (Thermo Scientific).

### *In silico* modelling of the possible interaction of relebactam with the Bla_Mab_

The Bla_Mab_ X-ray crystal structure was obtained from the Protein Data Bank, accession code 4YFM. The A chain was submitted to the GHECOM pocket-finding server (GHECOM 1.0)^33,34^. The top six pockets identified were used to define the target site for protein-ligand docking experiments between the enzyme and relebactam using CACHe Worksystem Pro (version 7.5.0.85, Fujitsu Ltd). The amino acid residues lining these pockets are given in Fig. 3(c). Pocket 1 corresponded to the main (catalytic) site in the enzyme and for the purposes of the docking experiment was redefined as all amino acid residues within 8 Å of serine 71. Hydrogen atoms were added using the default settings in line with presumed protonation states for ionisable amino acid side-chains. The positions of the added hydrogen atoms were optimised by locking the coordinates of all the non-hydrogen atoms and subjecting the system to a molecular mechanics (MM2) geometry optimisation. Relebactam was docked four times into each pocket, using CACHe Worksystem Pro. The amino acid side-chains in each pocket were allowed to be flexible as were all rotatable bonds in relebactam. The genetic algorithm settings for the docking protocol included population size 50, maximum generations 3000, crossover rate 0.8, mutation Rate 0.2 and convergence when the RMSD population fitness was less than 1. The best-scoring consensus complexes from each series of dockings were taken forward for molecular dynamics simulation. The required input files were prepared using the Antechamber module of the AMBER Tools package (Version 14^35^), implementing the ff14SB force field. The system was neutralised by addition of sodium ions and then solvated within a truncated octahedron of TIP3P water molecules extending 8 Å from the surface of the protein. Using the Amber 14 molecular dynamics package CUDA version^36–38^, the system was energy-minimised for 2,000 cycles using a non-bonded cut-off of 12 Å and then heated under constant volume to 300 K over 25 ps under Langevin dynamics (time step = 1 fs). The heating was continued at 300 K for at least 200 ns under constant pressure also using Langevin dynamics (SHAKE on, time step = 2 fs) using the Particle–Mesh–Ewald (PME) method to treat the long range electrostatic interactions with a 12 Å non-bonded cut-off.

## Supplementary information

Supplementary Dataset 1.

## Data Availability

All data will be made freely available by the authors upon request.
