# Author Correction: Effect of Amoxicillin in combination with Imipenem-Relebactam against *Mycobacterium abscessus*

**DOI:** 10.1038/s41598-020-61546-6

**Published:** 2020-03-06

**Authors:** Rose C. Lopeman, James Harrison, Daniel L. Rathbone, Maya Desai, Peter A. Lambert, Jonathan A. G. Cox

**Affiliations:** 1 School of Life and Health Sciences, Aston University, Aston Triangle, B4 7ET Birmingham, UK; 2 Birmingham Children’s Hospital, Birmingham Women’s and Children’s NHS Foundation Trust, Steelhouse Lane, B4 6NH Birmingham, UK

Correction to: *Scientific Reports* 10.1038/s41598-020-57844-8, published online 27 January 2020

The Acknowledgements section in this Article is incomplete.

“J.A.G.C. is grateful to the Academy of Medical Sciences, Global Challenges Research Fund and Birmingham Women’s and Children’s Hospital Charity Research Foundation (BWCHCRF) for their continued support of the Mycobacterial Research Group at Aston University. The Mycobacterium abscessus clinical isolates used in this study were provided by Dr. Simon Waddell, Brighton and Sussex Medical School. We are also grateful to Dr. Amit Chattopadhyay, Aston University, for technical support. This research was funded by Birmingham Women’s and Children’s Hospital Charity Research Foundation (BWCHCRF) (R.C.L. 50% PhD Studentship, match funded by Aston University Prize Scheme) and the Academy of Medical Sciences and Global Challenges Research Fund with a Springboard Grant (SBF003\1088:).”

should read:

“J.A.G.C. is grateful to the Academy of Medical Sciences, Global Challenges Research Fund and Birmingham Women’s and Children’s Hospital Charity Research Foundation (BWCHCRF) for their continued support of the Mycobacterial Research Group at Aston University. The Mycobacterium abscessus clinical isolates used in this study were provided by Dr. Simon Waddell, Brighton and Sussex Medical School. We are also grateful to Dr. Amit Chattopadhyay, Aston University, for technical support. This research was funded by Birmingham Women’s and Children’s Hospital Charity Research Foundation (BWCHCRF) (R.C.L. 50% PhD Studentship, match funded by Aston University Prize Scheme) and this project was supported by the Academy of Medical Sciences /the British Heart Foundation/the Government Department of Business, Energy and Industrial Strategy/ Global Challenges Research Fund /the Wellcome Trust Springboard Award [SBF003\1088].”

