# Supplementary material for: Effect of Amoxicillin in combination with Imipenem-Relebactam against Mycobacterium abscessus
Source: Sci Rep. 2020 Jan 27;10:928. doi: 10.1038/s41598-020-57844-8 (PMC6985242; doi:10.1038/s41598-020-57844-8)
Supplement: Supplementary file 1 — Supplementary Dataset 1. [file 41598_2020_57844_MOESM1_ESM.docx]

# **Supplementary Data**

**Effect of Amoxicillin in combination with Imipenem-Relebactam against *Mycobacterium abscessus***

Rose C. Lopeman^1†^, James Harrison^1†^, Daniel L. Rathbone^1^, Maya Desai^2^, Peter A. Lambert^1^ and Jonathan A. G. Cox^1^*

^1^School of Life and Health Sciences, Aston University, Aston Triangle, Birmingham, UK, B4 7ET

^2^Birmingham Children’s Hospital, Birmingham Women’s and Children’s NHS Foundation Trust, Steelhouse Lane, Birmingham, UK, B4 6NH

^†^These authors contributed equally to this work.

*Author to whom correspondence should be addressed:

Dr Jonathan A. G. Cox, School of Life and Health Sciences, Aston University, Aston Triangle, Birmingham, UK, B4 7ET; [J.a.g.cox@aston.ac.uk](mailto:J.a.g.cox@aston.ac.uk); 0(+44)121 204 5011

ORCID ID: 0000-0001-5208-4056

**Supplementary Figure 1: Overexpression of *M. abscessus* β-lactamase results in loss of relebactam-mediated sensitisation to amoxicillin.** A disk diffusion experiment and corresponding plate map demonstrating loss of sensitivity to amoxicillin and relebactam, and meropenem and relebactam in *M. abscessus* pVV16-*bla_Mab_* (S1a). This change in sensitivity can be attributed to the overexpression of Bla_Mab_ as the empty vector control (S1b) exhibited no change in sensitivity from the WT strain.

**Supplementary Figure 2: Novel Thin Layer Chromatography (TLC) assay exhibiting the activity of Bla_Mab_ in the turnover of penicillin V (high R_f_ value) to penicilloic acid (lower R_f_ value).** In the absence, or termination of activity of Bla_Mab_ (by boiling (100 °C for 1 h) or addition of known inhibitor avibactam (Lefebvre et al., 2017) (200 µg/mL) no lower R_f_ value spot corresponding to penicilloic acid is seen on the TLC plate. The addition of relebactam to the reaction between Bla_Mab_ and penicillin V also results in the absence of the lower Rf value spot, suggesting inhibition of Bla_Mab_. This inhibitory activity was seen within 10 seconds of pre-incubation of relebactam with Bla_Mab_, before addition of penicillin V, in a time course TLC assay. However, the addition of relebactam at the same time (t=0) as penicillin V resulted in a lack of inhibitory activity (S2a). The minimum concentration of relebactam required for inhibition of Bla_Mab_ in the TLC activity assay was assessed using a range of concentrations (200, 20, 2 and 0.2 µg/mL). Activity of Bla_Mab_ was maintained below a relebactam concentration of 2 µg/mL in the TLC activity assay, suggesting a minimal concentration of relebactam required to inhibit Bla_Mab_ in the assay is within the range of 20 to 2 µg/mL (S2b).
